# Periodontal Conditions and Whole Salivary IL-17A and -23 Levels among Young Adult *Cannabis sativa* (Marijuana)-Smokers, Heavy Cigarette-Smokers and Non-Smokers

**DOI:** 10.3390/ijerph17207435

**Published:** 2020-10-13

**Authors:** Fawad Javed, Abeer S. Al-Zawawi, Khaled S. Allemailem, Ahmad Almatroudi, Abid Mehmood, Darshan Devang Divakar, Abdulaziz A. Al-Kheraif

**Affiliations:** 1 Department of Orthodontics and Dentofacial Orthopedics, Eastman Institute for Oral Health, University of Rochester, Rochester, NY 14620, USA; fawjav@gmail.com; 2 Department of Periodontics and Community Dentistry, King Saud University, Riyadh 60169, Saudi Arabia; a.s.alzawawi1@gmail.com; 3 Department of Medical Laboratories, College of Applied Medical Sciences, Qassim University, Buraydah 51452, Saudi Arabia; k.allemailem@qu.edu.sa (K.S.A.); aamtrody@qu.edu.sa (A.A.); 4 Department of Dentistry, Postgraduate Medical Center, Karachi 75500, Pakistan; drabidmehmood12@gmail.com; 5 Dental Biomaterials Research Chair, Dental Health Department, College of Applied Medical Sciences, King Saud University, P.O. Box 10219, Riyadh 11433, Saudi Arabia; darshandevang@gmail.com

**Keywords:** alveolar bone loss, *Cannabis sativa*, cigarette smoking, marijuana, probing depth, whole saliva

## Abstract

In the United States, prevalence of marijuana-use has doubled in the past 2 decades. The aim was to compare the periodontal conditions and whole-salivary IL-17A and IL-23 levels among young adult marijuana-smokers, heavy cigarette-smokers and non-smokers. Self-reported marijuana-smokers, heavy-cigarette-smokers, non-smokers with periodontitis and periodontally-healthy non-smokers were included. Demographic data was recorded and full-mouth plaque index (PI), bleeding on probing (BoP), probing depth (PD) and clinical attachment loss (AL), marginal bone loss (MBL) and missing teeth were recorded. Levels of IL-17A and IL-23 levels were measured in the whole saliva. *p* < 0.01 was considered statistically significant. Fifteen-marijuana-smokers, 15 heavy-cigarette-smokers, 16 non-smokers-with-periodontitis and 15 periodontally-healthy-non-smokers) were included. The clinicoradiographic parameters were worse among marijuana-smokers (*p* < 0.01), cigarette-smokers (*p* < 0.01) and non-smokers-with-periodontitis (*p* < 0.01) than periodontally-healthy-non-smokers. Marijuana- and cigarette-smokers had Stage-IV/Grade C and non-smokers with periodontitis had Stage-III/Grade-C. Salivary IL-17A and IL-23 levels were higher in marijuana-smokers than cigarette-smokers (*p* < 0.01) and non-smokers-with-periodontitis (*p* < 0.01). Whole salivary IL-17A and IL-23 levels were higher among cigarette-smokers than non-smokers with periodontitis (*p* < 0.01) and periodontally-healthy-individuals (*p* < 0.01). Marijuana- and heavy cigarette-smokers have comparable clinicoradiographic periodontal statuses. This rejects hypothesis-1. However, whole salivary immunoinflammatory response may be moderately worse in marijuana-smokers compared with heavy cigarette-smokers and non-smoker with periodontitis thereby supporting hypothesis-2.

## 1. Introduction

Marijuana (*Cannabis sativa*), commonly known as bud, ganja, grass, pot and weed, is a grayish-green mix of dried flowers of *Cannabis sativa*; and is the most commonly used psychotropic drug in the United States after alcohol [1,2]. Tetrahydrocannabinol (THC), is the main chemical responsible for most of marijuana’s psychological effects including heightened sensory perception, sense of relaxation, laughter and increased appetite [3,4]. Moreover, Cannabinoids are anti-inflammatory agents by decreasing anti-oxidative activities and production of destructive-inflammatory cytokines and enhancing the induction of apoptosis and T-regulatory cells [5,6]. According to Hasin et al. [7], the prevalence of marijuana-use in the United States has doubled in the past 2 decades. Marijuana-use is more prevalent among males than females [1,2]; and its habitual use is associated disorders including but not limited to psychosis, pulmonary disorders and dependence/addiction [8]. Its use is more prevalent among males than females [1,2]. From a periodontal perspective, clinical studies [9,10] have shown that the mean number of sites per individual with a probing depth (PD) and clinical attachment loss (AL) of ≥4 mm are significantly higher among frequent marijuana-smokers compared with individuals not using recreational drugs.

The inflammatory immune response plays a role in the progression and etiopathogenesis of periodontitis [11,12,13]. Under periodontal inflammatory diseases, such as periodontitis, proinflammatory cytokines enhance soft tissue inflammation and marginal bone loss (MBL) [14,15]. Activation of Th17 cells produces interleukin (IL)-17A, a proinflammatory cytokine that induces inflammation and bone resorption by stimulating the release of matrix metalloproteinases and chemokines [16]. Moreover, other cytokines including interferon-gamma (IFN-γ), IL-1β and tumor necrosis factor-alpha (TNF-α) act synergistically to influence the effects of IL-17A [14,15,17]. Another cytokine, IL-23 that is produced by dendritic cells and macrophages plays a dominant role in IL-17A production [18]. It has been reported that IL-23 is associated with autoimmune destruction in diseases including allergic encephalomyelitis, arthritis and periodontitis [18,19,20]. In general, there is a clear consensus that expression of IL-17A and IL-23 in oral fluids (UWS and gingival crevicular fluid [GCF]) is associated with the etiopathogenesis of periodontitis [15,19,20,21]. Severity of periodontitis is worse in marijuana-smokers and individuals with a significantly higher daily frequency of smoking cigarettes compared with light-smokers (individuals smoking < 10 cigarettes daily) [9,10,22].

**Hypothesis** **1.** *Periodontal inflammatory parameters are worse among young adult marijuana-smokers and heavy cigarette-smokers compared with non-smokers with and without periodontitis.*

**Hypothesis** **2.** *Whole salivary immunoinflammatory response is worse among young adult marijuana-smokers and heavy cigarette-smokers compared with non-smokers with and without periodontitis.*

The aim was to compare the periodontal conditions and whole salivary IL-17A and IL-23 levels among young adult marijuana-smokers, heavy cigarette-smokers and non-smokers.

## 2. Materials and Methods

### 2.1. Ethical Approval

This study was approved by the human subject’s ethics board of Postgraduate Medical Center, Karachi, Pakistan (Approval # OS/DD/0157); and was conducted in accordance with the Helsinki declaration of 1975, as revised in 2013. All participants were aware that participation is completely voluntary and that withdrawal at any stage of the investigation bears no consequences. It was mandatory of individuals to have read and signed a written informed consent form. All participants were given verbal and written information about the significance of oral hygiene maintenance and deleterious effects of smoking on overall health. Moreover, all participants were also provided written information and contact details about “a tobacco/narcotic cessation program” that could help them quit the habit.

### 2.2. Inclusion and Exclusion Criteria

Self-reported heavy smokers (individuals who had at least 40 pack-years of smoking) [23,24]; marijuana-smokers (individuals who were daily smoking marijuana for at least 1 year) [25]; and non-smokers (individuals that reported to have never used any form of tobacco-product and/or recreational drug) were included [13,26]. Self-reported dual-smokers (individuals smoking cigarettes as well as marijuana), light and moderate smokers, patients with systemic diseases such as HIV+ individuals and patients with cardiovascular, hepatic, endocrine and/or renal diseases were excluded. Pregnant/lactating females, edentulous individuals and bilateral maxillary and mandibular third molars were not assessed. Moreover, patient that reported to have undergone periodontal treatment (such as ultrasonic scaling, scaling and root planing and/or guided bone or tissue regeneration procedures) and had used probiotics, antibiotics, steroids, cancer therapy and/or non-steroidal anti-inflammatory medications were excluded.

### 2.3. Questionnaire

Data related to age, gender, duration of cigarette-smoking (in pack-years) and marijuana smoking (in years) and mode of marijuana smoking (joint, spliff, and/or pot) was recorded. Participants were also asked about their highest level of education (school-, college- and university-level) they had attained. Individuals that had attained education from the Pakistani education system up to Grade-10 were categorized as having “school-level education” [27,28]. Individuals that had attained an additional of 2-years of education after graduation from school were categorized as having “college-level education” [28]. Individuals with University level education were defined as individuals who had graduated from a university [29]. Reason for smoking cigarettes and marijuana and family history of cigarette and marijuana smoking were also asked for. Patients were inquired if they had any interest and/or intention of quitting cigarette and marijuana smoking.

### 2.4. Clinicoradiographic Periodontal Parameters

In all patients full-mouth clinical AL [30], bleeding on probing (BOP) [31], PD [32] and plaque index (PI) [31] were measured by a skilled and standardized examiner (*Kappa* 0.85). These measurements were performed on 6 surfaces per tooth (midlingual/palatal, distolingual/palatal, mesiolingual/palatal, distobuccal, midbuccal and mesiobuccal). Number of missing teeth were recorded in all groups. Grossly-carious teeth with root remnants embedded in the jaw-bone were also considered missing. Using the long-cone paralleling-technique [33,34] digital intra-oral radiographs (Intra oral X-Ray-Systems—NOMAD/Pro-2 Gendex-Hatfield, PA, USA) were taken for all teeth. In all radiographs, marginal-bone-loss (MBL) was demarcated as the vertical void from 2-mm under the cement-enamel interface to the crest of interdental bone [35]. Radiographic assessments were performed by an experienced researcher (*Kappa* score 0.93) who was blinded to the study groups.

### 2.5. Periodontal Health and Staging and Grading of Periodontitis

Periodontal health was defined as a state of absence of the symptoms and signs of periodontal disease [36]; and periodontitis was defined using the following parameters: clinical AL of at least 1–2 mm, PD of ≥4 mm and horizontal MBL as described elsewhere [37].

### 2.6. Collection of Unstimulated Whole Saliva Samples and Determination of Whole Salivary IL-17A and IL-23 Levels

The UWS samples were collected by a calibrated researcher (*Kappa* 0.9) as described in previous studies [38,39]. In summary, all UWS samples were collected during early morning hours (between 7:00 a.m. and 8:00 a.m.) with the patients being in a fasting state. Patients were comfortably seated on a chair in a quiet room and requested to allow UWS to accumulate in the oral cavity over a 5-min duration. During this time, patients were advised to refrain from deglutition and jaw/lip movements. Patients were them requested to expectorate the UWS into a disposable plastic funnel, which was coupled with a gauged measuring-cylinder. The amount of expectorated UWS was recorded in milliliters. For each individual per group, unstimulated whole salivary flow rate (SFR) was determined in milliliters per minute (mL/min). The UWS samples were transferred to sterile plastic tubes with lid (^#^Fisherbrand™ Premium Microcentrifuge Tubes, Waltham, MA, USA) and centrifuged in a cold room at 3000 rpm for 5 min. The supernatant was collected and stored at −80° Celsius. All UWS samples were assessed for IL-17A and IL-23 levels within 24 h of collection.

### 2.7. Assessment of Whole Salivary IL-17A and -23 Levels

The IL-17A and IL-23 levels were assessed using a technique based on flow-cytometry (Luminex x-MAP technique, Luminex, Austin, TX, USA) as described in the study by Liukkonen et al. [15]. The supernatant was thawed and IL-17A and IL-23 levels were determined using standard kits (MILLIPLEX-map-kit, Human cytokine/chemokine panel, Millipore, Billerica, MA, USA) according to the guidelines provided by the manufacturer^††^. Whole salivary IL-23 and IL-17A concentrations were recorded in picograms per milliliter (pg/mL). The detection limits of IL-23 and IL-17A were 28.6 pg/mL and 0.2 pg/mL, respectively. The detection limits of IL-23 and IL-17A were 28.6 pg/mL and 0.2 pg/mL, respectively. Laboratory-based investigations were performed by a trained investigator (Kappa 0.91) who was blinded to the study groups.

### 2.8. Statistical Analysis

A computer-based statistical software (SPSS. Version 20, Chicago, IL, USA) was used to perform group comparisons in relation to clinicoradiographic parameters, SFR and whole salivary IL-17A and -23 levels. The Kolmogorov-Smirnov test was used to assess data normality. Group-wise, statistical evaluations were done using one-way-analysis-of variance and Bonferroni post-hoc adjustment tests. A *p*-value, which was less than 0.01 was designated as an indicator of statistical significance.

## 3. Results

### 3.1. General Characteristics of the Study Groups

In total, 15 marijuana-smokers, 15 heavy cigarette-smokers, 16 non-smokers with periodontitis and 15 periodontally-health non-smokers were included. All participants were male with no statistically significant difference in age. Marijuana-smokers were smoking marijuana as a “spliff” 10.6 ± 0.4 times daily for the past 11.1 ± 0.4 years. Cigarette-smoking had a smoking history of 43.6 ± 1.05 pack-years (36.5 ± 5.2 cigarettes per day). Graduate-level education was attained by 18.7% and 75% non-smokers with and without periodontitis, respectively. School-level education was the highest educational standard reported by 80% and 60% marijuana-smokers and heavy cigarette-smokers, respectively. Among cigarette-smokers, 73.3% and 26.7% individuals reported that they smoked cigarettes as the habit helped them manage psychological stress and concentrate, respectively. All marijuana-smokers reported that they used Cannabis to alleviate psychological stress. Tooth-brushing twice daily and flossing at least once daily was more often reported by non-smokers with a healthy periodontal status as compared to individuals in other groups (Table 1).

### 3.2. Clinicoradiographic Status and Staging/Grading of Periodontitis

The percentages of sites that presented with plaque accumulation, BoP, clinical AL and increased PD and MBL were significantly higher among marijuana-smokers (*p* < 0.01), cigarette-smokers (*p* < 0.01) and non-smokers with periodontitis (*p* < 0.01) compared with non-smokers without periodontitis. The numbers of missing teeth were significantly higher among marijuana-smokers (*p* < 0.01), cigarette-smokers (*p* < 0.01) and non-smokers with periodontitis (*p* < 0.01) compared with non-smokers without periodontitis. The numbers of missing teeth were higher among marijuana-smokers compared with non-smokers with periodontitis. Marijuana-smokers and cigarette-smokers had Stage IV/Grade C periodontitis and among non-smokers with periodontitis, the staging and grading of periodontitis was Stage III and Grade C, respectively (Table 2).

### 3.3. Salivary Flow Rate and Whole Salivary IL-17A and IL-23 Levels

There was no significant difference in the unstimulated whole SFR among marijuana-smokers, cigarette-smokers, non-smokers with periodontitis and periodontally-healthy non-smokers. Whole salivary IL-17A and IL-23 levels were significantly higher in marijuana-smokers compared with cigarette-smokers (*p* < 0.01) and non-smokers with periodontitis (*p* < 0.01). Whole salivary IL-17A and IL-23 levels were significantly higher among cigarette-smokers compared with non-smokers with periodontitis (*p* < 0.01) and periodontally-healthy individuals (*p* < 0.01) (Table 3).

## 4. Discussion

From a clinicoradiographic perspective, the present results showed that periodontal inflammatory status was worse in marijuana-smokers and cigarette-smokers compared with non-smokers without periodontitis (positive controls). These results are in accordance with previous clinical studies [9,13]. One justification for this is that nicotine (a major and addictive component in tobacco) induces destructive effects on human gingival fibroblasts (HGF), periodontal ligament cells and alveolar bone [40,41]. Moreover, results from an in-vitro study [42] showed that exposure of HGF to high concentrations of nicotine and cotinine (a metabolite of nicotine) impairs their attachment to root surfaces. With reference to detrimental effects of marijuana-smoking on alveolar bone, results from an experimental study in rat-models showed that marijuana-smoke inhalation increases alveolar bone loss and decreases bone density [43]. According to Nakajima et al. [44] cannabinoids possibly contribute towards the etiopathogenesis of periodontitis via expression of cannabinoid receptors in periodontal tissues. The present results no significant difference in clinical and radiographic periodontal inflammatory parameters among heavy cigarette-smokers and marijuana-smokers. This was in contradiction to the proposed hypothesis. It is noteworthy that in the present study, marijuana was smoked as a “spliff,” which is a combination of marijuana and tobacco wrapped in a thin paper. The authors speculate that both marijuana and nicotine induce comparable levels of periodontal destruction; and hence nicotine exposure during heavy cigarette-smoking induces clinicoradiographic periodontal inflammatory effects that are similar to using marijuana as a “spliff.” However, this is merely a speculation and further studies are warranted to confirm this hypothesis. Despite the clinicoradiographic similarities among cigarette- and marijuana-smokers, a marked variation in the expression of proinflammatory cytokines in the UWS was noted in these individuals. The present results showed that whole salivary IL-17A and IL-23 levels were significantly higher in the UWS of marijuana-smokers compared with non-smokers with periodontitis. This suggests that whole salivary assessment of IL-17A and IL-23 can be used as a biomarker of periodontitis especially among tobacco-smokers and recreational-drug smokers. Our results further demonstrate that on a molecular level, the deleterious effects of smoking marijuana as a “spliff” are worse than smoking cigarettes. However, by no means does this imply that cigarette-smoking is safer than smoking Cannabis. The authors performed a regression analysis to corelate the clinicoradiographic parameters and whole salivary IL-17 and IL-23 levels with age, gender education status and frequency of smoking cigarettes and marijuana; however, no statistically significant association between these parameters existed (data not shown).

In the present study, exclusively heavy cigarette-smokers were included. A reasoning for this criterion was based on the perception that since marijuana jeopardizes HGF, periodontal ligament cells as well as alveolar bone [40,41]; comparison of marijuana-smokers with light- and moderate cigarette-smokers may give false-positive outcomes. However, there is a lack of consensus regarding the precise classification of cigarette-smokers based upon smoking history (pack-years). For instance, in the study by Hassan et al. [45] heavy-smokers were defined as individuals who reported over 20 pack-years of smoking; whereas in the studies by Langevin et al. [46] and Karlsson et al. [47] heavy-smokers were defined as individuals who had a smoking history of more than 18.3 and 60 pack-years, respectively. In the present study, individuals with a cigarette-smoking history of at least 40 pack-years were classified as “heavy” cigarette-smokers as this criterion seems to be a rather median value for the wide range pack-year based (~18 to ~60 pack-years) definitions used to define “heavy-smokers.” The definition of heavy-smokers used in the present observational study is in consistence with the study by Costa et al. [23]; however, there is a dire need to implicate and adopt standardized criteria for definition of “light”, “moderate” 78 and “heavy” cigarette smokers that can be implicated globally for clinical and biomedical research purposes. It is further suggested that criteria for classification for recreational-drug (such a marijuana-smokers) users based upon recreational-drug use history may also be useful for future studies in the related field.

Determination of the optimal sample-size for a study is critical as it helps estimate the power to detect statistical significances [48]. In this regard, a major limitation of the present study is that a prior sample-size estimation (power analysis was not performed. Therefore, the reported *p*-value for group-comparisons should be cautiously interpreted. Moreover, education status was poorer among marijuana- and heavy cigarette-smokers compared with non-smokers with periodontitis and periodontally-healthy controls. Interdental flossing and routine visits to oral healthcare providers were more often reported by periodontally-healthy individuals compared with patients in other groups. These factors could have contributed towards worsening periodontal inflammation among marijuana-smokers, heavy cigarette-smokers and non-smokers with periodontitis. It is also noteworthy that tobacco-smoking was self-reported in the present study. However, a true determination of a person being a smoker or a passive/non-smoker can be done using biological investigations such as assessment of whole salivary cotinine levels [11]. Furthermore, based upon the staging and grading criteria, periodontal inflammation was worse in marijuana-and cigarette smokers compared with non-smokers with periodontitis. It is speculated that non-smokers with a staging and grading of periodontitis may demonstrate IL-17 and IL-23 levels that are comparable with cigarette-smokers and marijuana-smokers. At least 73% of the heavy-cigarette-smokers and all (100%) marijuana-smokers reported that they achieved a state of mind referred to as “stress relief” after using their respective products. It is possible that these individuals had an underprivileged socioeconomic status that could be associated with their poor education status. This could have compelled these individuals to use tobacco (cigarettes as in the present scenario) and cannabis to temporarily alleviate socioeconomic-based stresses. Patient health education and anti-tobacco and cannabis based programs may play a role in improving the quality of life of susceptible populations. Further studies are needed to test these hypotheses.

## 5. Conclusions

Marijuana- and heavy cigarette-smokers have comparable clinicoradiographic periodontal statuses. This rejects hypothesis-1. However, whole salivary immunoinflammatory response may be moderately worse in marijuana-smokers compared with heavy cigarette-smokers and non-smoker with periodontitis thereby supporting hypothesis-2.

## Figures and Tables

**Table 1:** Characteristics of the study cohort.

Parameters	Marijuana-Smokers	Cigarette-Smokers	Non-Smokers with Periodontitis	Periodontally-Healthy Non-Smokers
Number of Individuals (*n*)	15	15	16	15
Gender (male)	15	15	16	15
Age in Years (mean ± SD)	38.3 ± 0.5 years	40.2 ± 0.5 years	38.4 ± 0.7 years	40.3 ± 0.4 years
Duration of Marijuana-Smoking in Years (mean ± SD)	11.1 ± 0.4 years	NA	NA	NA
Daily Frequency of Marijuana-Smoking (times/day)	10.6 ± 0.4 times daily	NA	NA	NA
Duration of Cigarette-Smoking in Pack-years (mean ± SD)	NA	43.6 ± 1.05 pack years	NA	NA
Education Status				
School-level (*n*) (%)	12 (80%)	9 (60%)	6 (37.5%)	0
College-level (*n*) (%)	3 (20%)	6 (40%)	7 (43.8%)	3 (25%)
University-level (*n*) (%)	0	0	3 (18.7%)	12 (75%)
Reasons for Marijuana or Cigarette Smoking				
Stress-relief	11 (73.3%)	15 (100%)	NA	NA
Helps with Concentration	4 (26.7%)	0	NA	NA
No reason	0	0	NA	NA
Most recent visit to dentist/hygienist				
Within 6 months	0	0	0	0
Within 6–12-months	0	0	0	9 (60%)
Over 1-year ago	0	0	3 (18.7%)	4 (26.7%)
Over 2-years ago	1 (6.7%)	9 (60%)	9 (56.3%)	2 (13.3%)
Over 3 years ago	2 (13.3%)	6 (40%)	2 (12.5%)	0
Don’t remember	12 (80%)	0	2 (12.5%)	0
Daily Toothbrushing				
Once daily	12 (80%)	9 (60%)	11 (68.8%)	13 (86.7%)
Twice daily	3 (20%)	6 (40%)	5 (31.2%)	2 (13.3%)
Flossing at least once Daily	0	0	0	5 (33.3%)

NA: Not applicable. SD: Standard deviation.

**Table 2:** Clinicoradiographic parameters and staging/grading of periodontitis.

Parameters (Mean ± SD)	Marijuana-Smokers (*n* = 15)	Cigarette-Smokers (*n* = 15)	Non-Smokers with Periodontitis (*n* = 16)	Periodontally-Healthy Non-Smokers (*n* = 15)
Plaque Index	69.3 ± 8.2%	63.4 ± 6.2%	60.5 ± 9.1%	10.3 ± 6.4% *
Bleeding on Probing	30.5 ± 4.8% ^†^	27.3 ± 5.1% ^†^	69.4 ± 12.2%	9.6 ± 2.5% *
Probing Depth	7.1 ± 0.3 mm	6.8 ± 0.2 mm	4.5 ± 0.3 mm	0.6 ± 0.08 mm *
Clinical Attachment Loss	6.2 ± 0.3 mm	5.8 ± 0.2 mm	4.3 ± 0.2 mm	0.2 ± 0.02 mm *
Marginal Bone Loss ^(a)^ (mesial)	7.2 ± 0.5 mm	6.2 ± 0.4 mm	4.9 ± 0.3 mm	0.2 ± 0.05 mm *
Marginal Bone Loss ^(a)^ (distal)	7.4 ± 0.3 mm	6.08 ± 0.6 mm	5.1 ± 0.4 mm	0.2 ± 0.06 mm *
Missing Teeth	14.2 ± 2.3 teeth	10.5 ± 1.3 teeth	7.1 ± 0.3 teeth ^‡^	1.3 ± 0.04 teeth *
Staging/Grading	Stage IV/Grade C	Stage IV/Grade C	Stage III/Grade C	NA

^(a)^ Horizontal marginal bone loss extending to at least the mid-root length. NA: Not applicable. * Compared with marijuana-smokers (*p* < 0.01), cigarette-smokers (*p* < 0.01) and non-smokers with periodontitis (*p* < 0.01). ^†^ Compared with non-smokers with- (*p* < 0.01) and without (*p* < 0.01) periodontitis. ^‡^ Compared with marijuana-smokers (*p* < 0.01).

**Table 3:** Whole salivary flow rate and IL-17A and IL-23 levels.

Parameters	Marijuana-Smokers (*n* = 15) (Mean ± SD)	Cigarette-Smokers (*n* = 15) (Mean ± SD)	Non-Smokers with Periodontitis (*n* = 16) (Mean ± SD)	Periodontally-Healthy Non-Smokers (*n* = 15) (Mean ± SD)
SFR (mL/min)	0.34 ± 0.01 mL/min *	0.33 ± 0.01 mL/min	0.35 ± 0.05 mL/min	0.37 ± 0.02 mL/min
IL-17A (pg/mL)	27.3 ± 5.3 pg/mL ^†^	13.4 ± 1.2 pg/mL ^§^	5.2 ± 1.3 pg/mL ^¶^	0.4 ± 0.008 pg/mL
IL-23 (pg/mL)	239.4 ± 8.1 pg/mL ^‡^	125.2 ± 8.6 pg/mL ^‖^	66.1 ± 7.4 pg/mL ^#^	30.4 ± 0.05 pg/mL

IL: Interleukin. SFR: Salivary flow rate. * Compared with cigarette-smokers (*p* < 0.01) and non-smokers with periodontitis (*p* < 0.01); ^†^ Compared with cigarette-smokers (*p* < 0.01) and non-smokers with periodontitis (*p* < 0.01); ^‡^ Compared with cigarette-smokers (*p* < 0.01) and non-smokers with periodontitis (*p* < 0.01); ^§^ Compared with non-smokers with periodontitis (*p* < 0.01); ^‖^ Compared with non-smokers with periodontitis (*p* < 0.01) and periodontally-healthy individuals (*p* < 0.01); ^¶^ Compared with periodontally-healthy individuals (*p* < 0.01); ^#^ Compared with periodontally-healthy individuals (*p* < 0.01).
